# *N*-[(Morpholin-4-yl)carbonothio­yl]-4-nitro­benzamide

**DOI:** 10.1107/S1600536810018763

**Published:** 2010-05-22

**Authors:** Sohail Saeed, Rashid Mehmood, Wing-Tak Wong, Ghulam Waris, Abdul Manan

**Affiliations:** a Department of Chemistry, Research Complex, Allama Iqbal Open University, Islamabad, Pakistan; b Department of Applied Biology and Chemical Technology, The Hong Kong Polytechnic University, Hung Hom, Kowloon, Hong Kong SAR, People’s Republic of China; c National Engineering & Scientific Commission, PO Box 2801, Islamabad, Pakistan

## Abstract

In the title compound, C_12_H_13_N_3_O_4_S, the nitro group is slightly twisted [6.58 (11)°] from the benzene ring plane. The morpholine ring adopts a chair form. In the crystal, inter­molecular N—H⋯O hydrogen bonds link the mol­ecules into chains along [110]. There are also π–π contacts [centroid–centroid distance = 3.8301 (11) Å] and C—H⋯π inter­actions to stack neighbouring benzene rings and link the chains into a three-dimensional network. C—H⋯O and C—H⋯S inter­actions are also observed.

## Related literature

For the use of thio­urea derivatives in the analysis of transition metals, see: Arslan *et al.* (2003 ▶). For the biological and agrochemical activity of thio­ureas and their transition metal complexes, see: Saeed *et al.* (2008 ▶, 2009 ▶, 2010 ▶); Che *et al.* (1999 ▶); Saeed & Parvez (2005 ▶). For their catalytic properties, see: Gu *et al.* (2007 ▶). For thio­ureas as ligands in coordination chemistry, see: Burrows *et al.* (1999 ▶); Henderson *et al.* (2002 ▶); Schuster *et al.* (1990 ▶).
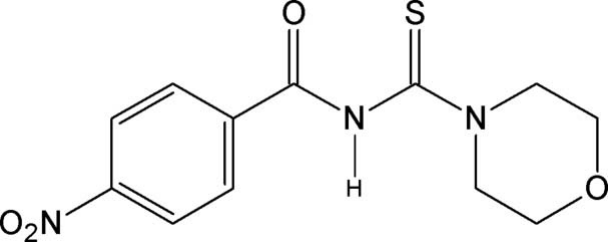


## Experimental

### 

#### Crystal data

C_12_H_13_N_3_O_4_S
*M* *_r_* = 295.31
Triclinic, 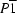

*a* = 6.9867 (11) Å
*b* = 7.4047 (11) Å
*c* = 14.261 (2) Å
α = 88.654 (2)°
β = 82.805 (2)°
γ = 65.638 (2)°
*V* = 666.46 (18) Å^3^
*Z* = 2
Mo *K*α radiation
μ = 0.26 mm^−1^
*T* = 298 K
0.23 × 0.20 × 0.08 mm

#### Data collection

Bruker SMART 1000 CCD diffractometer
Absorption correction: multi-scan (*SADABS*; Sheldrick, 1996 ▶) *T*_min_ = 0.943, *T*_max_ = 0.980
4478 measured reflections
2916 independent reflections
2485 reflections with *I* > 2σ(*I*)
*R*_int_ = 0.009

#### Refinement

*R*[*F* ^2^ > 2σ(*F* ^2^)] = 0.037
*wR*(*F* ^2^) = 0.106
*S* = 1.09
2916 reflections
186 parameters
H atoms treated by a mixture of independent and constrained refinement
Δρ_max_ = 0.34 e Å^−3^
Δρ_min_ = −0.31 e Å^−3^

### 

Data collection: *SMART* (Bruker, 1996 ▶); cell refinement: *SAINT* (Bruker, 2006 ▶); data reduction: *SAINT* and *CrystalStructure* (Rigaku/MSC and Rigaku, 2006 ▶); program(s) used to solve structure: *SHELXS97* (Sheldrick, 2008 ▶); program(s) used to refine structure: *SHELXL97* (Sheldrick, 2008 ▶); molecular graphics: *ORTEPII* (Johnson, 1976 ▶); software used to prepare material for publication: *SHELXL97*.

## Supplementary Material

Crystal structure: contains datablocks global, I. DOI: 10.1107/S1600536810018763/sj2785sup1.cif

Structure factors: contains datablocks I. DOI: 10.1107/S1600536810018763/sj2785Isup2.hkl

Additional supplementary materials:  crystallographic information; 3D view; checkCIF report

## Figures and Tables

**Table 1:** Hydrogen-bond geometry (Å, °) *Cg*1 is the centroid of the C1–C6 ring.

*D*—H⋯*A*	*D*—H	H⋯*A*	*D*⋯*A*	*D*—H⋯*A*
N2—H2*N*⋯O4^i^	0.82 (2)	2.27 (2)	3.0947 (17)	178.1 (18)
C3—H3⋯O1^ii^	0.93	2.43	3.206 (2)	141
C6—H6⋯O3^iii^	0.93	2.65	3.377 (2)	135
C9—H9*B*⋯O4^iv^	0.97	2.67	3.590 (2)	159
C10—H10*A*⋯S1^v^	0.97	2.98	3.7913 (18)	142
C12—H12*B*⋯S1^vi^	0.97	2.97	3.7196 (18)	135
C2—H2⋯*Cg*1^vii^	0.93	3.45	3.682 (2)	83

Symmetry codes: (i) 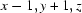; (ii) 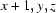; (iii) 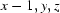; (iv) 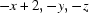; (v) 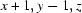; (vi) 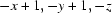; (vii) 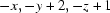.

**Fig. 1.**
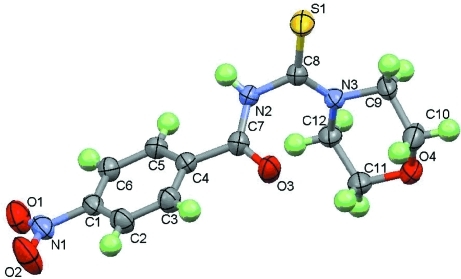
The structure of the title compound with 50% probability thermal ellipsoids and the atom numbering scheme.

**Fig. 2.**
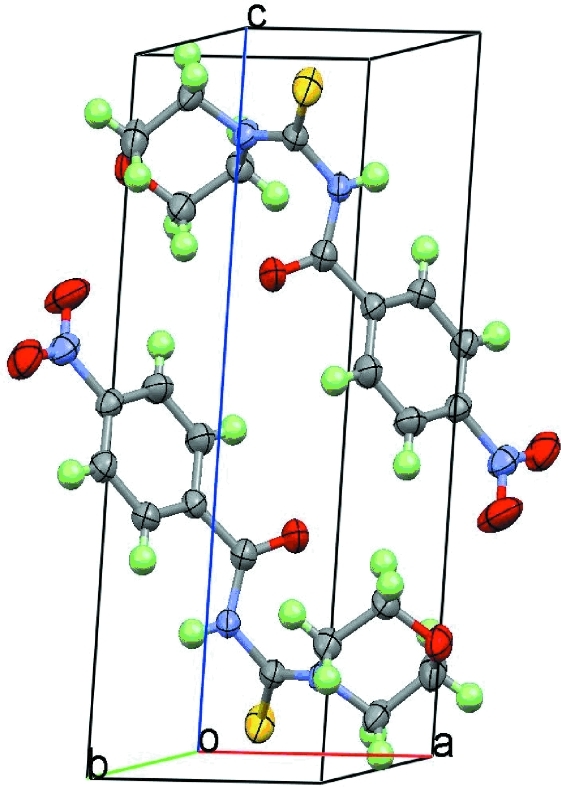
The unit cell packing diagram of the compound.

**Fig. 3.**
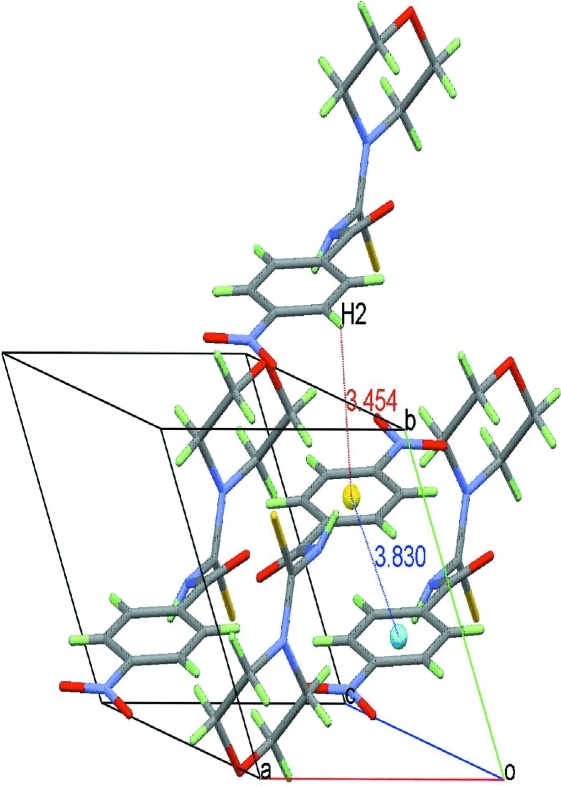
A capped-stick diagram of the unit cell showing π–π and C–H···π interactions. The yellow sphere indicates the centroid of the C1—C6 phenyl ring, and the cyan sphere represents the centroid (C1—C6)i (i symmetry code: -x, 1-y, 1-z).

## supplementary crystallographic information

### Comment

Cobalt, nickel and copper are essential elements for biological systems and are present in trace quantities. In each case, trace analysis of these elements requires pre-concentration prior to their analysis.  Thiourea derivatives are selective analytical reagents, especially for the determination of transition metals in complex inferring matrices (Arslan *et al.*, 2003). The biological activity of complexes with thiourea derivatives has been successfully screened for various biological actions. Thioureas and their transition metal complexes are found to exhibit a wide range of biological activities including anticancer (Saeed *et al.*, 2010), antifungal (Saeed *et al.*, 2008), antiviral, antibacterial (Saeed *et al.*, 2009), anti-tubercular, anti-thyroidal, herbicidal and insecticidal activities, organocatalyst (Gu *et al.*, 2007).  Thioureas also have a long history as ligands in coordination chemistry and coordinate to a metal via sulfur and oxygen (Burrows *et al.*, 1999). These hard and soft donor atoms provide a multitude of bonding possibilities (Henderson *et al.*, 2002). Hydrogen bonding behavior of some thioureas has been investigated and it is found that intramolecular hydrogen bonds between the carbonyl oxygen and a hydrogen atom on N' is common. The complexing capacity of thiourea derivatives has been reported in several papers (Schuster *et al.*, 1990). Some acyl thioureas have been found to possess pesticidal activities and promote plant growth (Saeed & Parvez, 2005) while some have been shown to have notable positive effect on the germination of maize seeds as well as on the chlorophyll contents in seedling leaves (Che *et al.*, 1999). With the simultaneous presence of S, N and O electron donors, the versalitility and interesting behavior of acylthioureas as building blocks in polydentate ligands for metal ions have become a topic of interest in the last few years.It has been reported that substituted acylthiourea ligands might act as monodentate sulfur donors, bidentate oxygen and nitrogen donors. As a part of our continuing interest in biologically active thiourea derivatives and their transition metal complexes, we are reporting a route for synthesis of these compounds by using tetrabutylammonium bromide (TBAB) as a phase transfer catalyst (PTC) to augment the yield of products. In view of the above and in continuation of our research program concerned with structural modification of certain biologically active thiourea derivatives and their transition metal complexes with the purpose of enhancing their biological activity, we aimed to incorporate the aliphatic and aromatic moieties in the substituted phenyl nucleus with thiourea functionality to obtain new functions in an attempt to improve the antimicrobial profile of compounds. The compound, *N*-(morpholine-4-carbothioyl)-4-nitro-benzamide, crystallizes in a triclinic primitive space group, P -1 (#2). Like its other analogue, the molecule is not planar. The nitro group, N1/O1/O2, is slightly twisted (6.58 (11)°) from the benzene ring plane of C1—C6. The thioureido group is also twisted. The amide group C4/C7/O3/N2 is making a dihedral angle of 25.68 (5)° with the benzene ring plane and 65.10 (6)° with the thiourea group, N2/C8/S1/N3. The morpholine ring is in the chair form.

Inter-molecular N2—H2N···O4 H-bond interactions link the molecules to form 1-dimensional chains in the [110] plane in the crystal lattice Table 1. π–π interactions help to stack the chains into 3-D network. The centroid-to-centroid distance of the ring C1—C6 and (C1—C6)^i^ (^i^ symmetry code: -x, 1-y, 1-z) 3.8301 (11) and the perpendicular distance between the centriod of C1—C6 and mean plane of (C1—C6)^i^ is 3.449 Å. C—H···π interactions are also obseved with the distance between H2^ii^ and centroid of C1—C6  3.454Å (^ii^ symmetry code: -x, 2-y, 1-z) Table 1.

### Experimental

All the chemicals used for the preparation were of reagent grade quality. The ammonium thiocyanate was dried by heating at 100°C and the acetone using potassium carbonate. A solution of 4-nitrobenzoyl chloride (0.01 mol) in anhydrous acetone (80 ml) and 3% tetrabutylammonium bromide (TBAB) as a phase transfer catalyst (PTC) in anhydrous acetone was added dropwise to a suspension of dry ammonium thiocyanate (0.01 mol) in acetone (50 ml) and the reaction mixture was refluxed for 45 min. After cooling to room temperature, a solution of morpholine (0.01 mol) in anhydrous acetone (25 ml) was added dropwise and the resulting mixture refluxed for 3 h. Hydrochloric acid (0.1 M, 400 ml) was added, and the solution was filtered. The solid product was washed with water and purified by re-crystallization from an ethanol-dichloromethane mixture (1:2).

### Refinement

All of the C-bound H atoms are observable from difference Fourier map but are all placed at geometrical positions with C—H = 0.93 and 0.97Å for phenyl and methylene H-atoms. All C-bound H-atoms are refined using riding model with *U*_iso_(H) = 1.2*U*_eq_(Carrier).

The N-bound H atoms are located from difference Fourier map and refined isotropically.

Highest peak is 0.34 at (0.6632, 0.7234, 0.1221) [0.91Å from S1] Deepest hole is -0.31 at (0.5416, 0.7531, 0.0302) [0.71Å from S1]

### Crystal data

**Table:**

C_12_H_13_N_3_O_4_S	*Z* = 2
*M**_r_* = 295.31	*F*(000) = 308
Triclinic, *P*1	*D*_x_ = 1.472 Mg m^−^^3^
Hall symbol: -P 1	Melting point: 445 K
*a* = 6.9867 (11) Å	Mo *K*α radiation, λ = 0.71073 Å
*b* = 7.4047 (11) Å	Cell parameters from 4479 reflections
*c* = 14.261 (2) Å	θ = 2.9–27.5°
α = 88.654 (2)°	µ = 0.26 mm^−^^1^
β = 82.805 (2)°	*T* = 298 K
γ = 65.638 (2)°	Prism, yellow
*V* = 666.46 (18) Å^3^	0.23 × 0.20 × 0.08 mm

### Data collection

**Table:**

Bruker SMART 1000 CCD diffractometer	2916 independent reflections
Radiation source: fine-focus sealed tube	2485 reflections with *I* > 2σ(*I*)
graphite	*R*_int_ = 0.009
ω scans	θ_max_ = 27.5°, θ_min_ = 2.9°
Absorption correction: multi-scan (*SADABS*; Sheldrick, 1996)	*h* = −9→9
*T*_min_ = 0.943, *T*_max_ = 0.980	*k* = −9→8
4478 measured reflections	*l* = −14→18

### Refinement

**Table:**

Refinement on *F*^2^	Primary atom site location: structure-invariant direct methods
Least-squares matrix: full	Secondary atom site location: difference Fourier map
*R*[*F*^2^ > 2σ(*F*^2^)] = 0.037	Hydrogen site location: inferred from neighbouring sites
*wR*(*F*^2^) = 0.106	H atoms treated by a mixture of independent and constrained refinement
*S* = 1.09	*w* = 1/[σ^2^(*F*_o_^2^) + (0.0533*P*)^2^ + 0.1461*P*] where *P* = (*F*_o_^2^ + 2*F*_c_^2^)/3
2916 reflections	(Δ/σ)_max_ < 0.001
186 parameters	Δρ_max_ = 0.34 e Å^−^^3^
0 restraints	Δρ_min_ = −0.31 e Å^−^^3^

### Special details

**Table:**

Geometry. All esds (except the esd in the dihedral angle between two l.s. planes) are estimated using the full covariance matrix. The cell esds are taken into account individually in the estimation of esds in distances, angles and torsion angles; correlations between esds in cell parameters are only used when they are defined by crystal symmetry. An approximate (isotropic) treatment of cell esds is used for estimating esds involving l.s. planes.
Refinement. Refinement of *F*^2^ against ALL reflections. The weighted *R*-factor wR and goodness of fit *S* are based on *F*^2^, conventional *R*-factors *R* are based on *F*, with *F* set to zero for negative *F*^2^. The threshold expression of *F*^2^ > 2σ(*F*^2^) is used only for calculating *R*-factors(gt) etc. and is not relevant to the choice of reflections for refinement. *R*-factors based on *F*^2^ are statistically about twice as large as those based on *F*, and *R*- factors based on ALL data will be even larger.

### Fractional atomic coordinates and isotropic or equivalent isotropic displacement parameters (Å^2^)

**Table:**

	*x*	*y*	*z*	*U*_iso_*/*U*_eq_	
S1	0.59857 (7)	0.73600 (6)	0.06951 (4)	0.05471 (16)	
O1	−0.4619 (2)	0.8315 (2)	0.56951 (10)	0.0682 (4)	
O2	−0.2725 (3)	0.8900 (3)	0.66055 (10)	0.0896 (6)	
O3	0.58764 (17)	0.5059 (2)	0.32004 (8)	0.0563 (3)	
O4	0.98995 (18)	−0.01437 (17)	0.15537 (9)	0.0531 (3)	
N1	−0.2970 (2)	0.8351 (2)	0.58493 (10)	0.0491 (3)	
N2	0.3781 (2)	0.6145 (2)	0.20248 (9)	0.0393 (3)	
N3	0.67667 (19)	0.36725 (18)	0.12218 (9)	0.0385 (3)	
C1	−0.1170 (2)	0.7713 (2)	0.50874 (10)	0.0392 (3)	
C2	0.0756 (3)	0.7565 (3)	0.52983 (11)	0.0480 (4)	
H2	0.0917	0.7861	0.5906	0.058*	
C3	0.2441 (3)	0.6969 (3)	0.45888 (12)	0.0466 (4)	
H3	0.3762	0.6839	0.4720	0.056*	
C4	0.2183 (2)	0.6559 (2)	0.36783 (10)	0.0370 (3)	
C5	0.0231 (2)	0.6692 (2)	0.34882 (11)	0.0404 (3)	
H5	0.0064	0.6394	0.2882	0.049*	
C6	−0.1473 (2)	0.7268 (2)	0.42000 (11)	0.0423 (3)	
H6	−0.2785	0.7352	0.4081	0.051*	
C7	0.4116 (2)	0.5862 (2)	0.29591 (11)	0.0404 (3)	
C8	0.5562 (2)	0.5601 (2)	0.13166 (10)	0.0366 (3)	
C9	0.8852 (3)	0.2872 (2)	0.06511 (12)	0.0491 (4)	
H9A	0.9256	0.3943	0.0456	0.059*	
H9B	0.8801	0.2192	0.0088	0.059*	
C10	1.0458 (3)	0.1439 (3)	0.12361 (15)	0.0562 (5)	
H10A	1.1839	0.0895	0.0860	0.067*	
H10B	1.0554	0.2148	0.1779	0.067*	
C11	0.7884 (3)	0.0630 (2)	0.21261 (12)	0.0484 (4)	
H11B	0.7980	0.1283	0.2689	0.058*	
H11A	0.7507	−0.0456	0.2325	0.058*	
C12	0.6179 (2)	0.2085 (2)	0.16003 (12)	0.0428 (3)	
H12B	0.5951	0.1395	0.1085	0.051*	
H12A	0.4864	0.2658	0.2024	0.051*	
H2N	0.275 (3)	0.715 (3)	0.1911 (13)	0.049 (5)*	

### Atomic displacement parameters (Å^2^)

**Table:**

	*U*^11^	*U*^22^	*U*^33^	*U*^12^	*U*^13^	*U*^23^
S1	0.0490 (3)	0.0384 (2)	0.0673 (3)	−0.01365 (18)	0.0083 (2)	0.01329 (19)
O1	0.0487 (7)	0.0877 (10)	0.0656 (9)	−0.0304 (7)	0.0118 (6)	−0.0065 (7)
O2	0.0829 (11)	0.1409 (16)	0.0466 (8)	−0.0539 (11)	0.0186 (7)	−0.0273 (9)
O3	0.0357 (6)	0.0730 (8)	0.0458 (6)	−0.0071 (5)	−0.0071 (5)	−0.0031 (6)
O4	0.0470 (6)	0.0382 (6)	0.0582 (7)	−0.0045 (5)	0.0022 (5)	0.0077 (5)
N1	0.0521 (8)	0.0472 (8)	0.0425 (8)	−0.0190 (6)	0.0078 (6)	0.0018 (6)
N2	0.0313 (6)	0.0388 (7)	0.0378 (7)	−0.0060 (5)	−0.0005 (5)	0.0071 (5)
N3	0.0367 (6)	0.0339 (6)	0.0387 (7)	−0.0110 (5)	0.0033 (5)	0.0041 (5)
C1	0.0417 (8)	0.0345 (7)	0.0361 (7)	−0.0129 (6)	0.0034 (6)	0.0033 (6)
C2	0.0521 (9)	0.0562 (10)	0.0348 (8)	−0.0219 (8)	−0.0031 (7)	−0.0040 (7)
C3	0.0400 (8)	0.0562 (10)	0.0431 (9)	−0.0190 (7)	−0.0052 (6)	−0.0015 (7)
C4	0.0365 (7)	0.0335 (7)	0.0357 (7)	−0.0098 (6)	−0.0024 (6)	0.0040 (6)
C5	0.0405 (8)	0.0424 (8)	0.0350 (7)	−0.0136 (6)	−0.0055 (6)	0.0029 (6)
C6	0.0365 (7)	0.0452 (8)	0.0433 (8)	−0.0156 (6)	−0.0024 (6)	0.0030 (6)
C7	0.0361 (7)	0.0392 (8)	0.0404 (8)	−0.0108 (6)	−0.0026 (6)	0.0003 (6)
C8	0.0327 (7)	0.0374 (7)	0.0361 (7)	−0.0116 (6)	−0.0026 (5)	0.0037 (6)
C9	0.0453 (8)	0.0399 (8)	0.0475 (9)	−0.0086 (7)	0.0137 (7)	0.0034 (7)
C10	0.0402 (8)	0.0441 (9)	0.0723 (12)	−0.0089 (7)	0.0038 (8)	0.0069 (8)
C11	0.0491 (9)	0.0414 (8)	0.0457 (9)	−0.0117 (7)	−0.0005 (7)	0.0094 (7)
C12	0.0427 (8)	0.0367 (8)	0.0462 (8)	−0.0153 (6)	−0.0011 (6)	0.0061 (6)

### Geometric parameters (Å, °)

**Table:**

S1—C8	1.6635 (15)	C3—C4	1.390 (2)
O1—N1	1.211 (2)	C3—H3	0.9300
O2—N1	1.217 (2)	C4—C5	1.387 (2)
O3—C7	1.2157 (19)	C4—C7	1.498 (2)
O4—C10	1.428 (2)	C5—C6	1.389 (2)
O4—C11	1.4302 (19)	C5—H5	0.9300
N1—C1	1.4753 (19)	C6—H6	0.9300
N2—C7	1.378 (2)	C9—C10	1.514 (3)
N2—C8	1.4219 (18)	C9—H9A	0.9700
N2—H2N	0.82 (2)	C9—H9B	0.9700
N3—C8	1.3249 (19)	C10—H10A	0.9700
N3—C9	1.4660 (19)	C10—H10B	0.9700
N3—C12	1.4682 (19)	C11—C12	1.506 (2)
C1—C2	1.375 (2)	C11—H11B	0.9700
C1—C6	1.378 (2)	C11—H11A	0.9700
C2—C3	1.378 (2)	C12—H12B	0.9700
C2—H2	0.9300	C12—H12A	0.9700
			
C10—O4—C11	110.05 (12)	O3—C7—C4	120.79 (14)
O1—N1—O2	123.01 (14)	N2—C7—C4	116.60 (13)
O1—N1—C1	118.84 (14)	N3—C8—N2	114.93 (13)
O2—N1—C1	118.15 (15)	N3—C8—S1	125.69 (11)
C7—N2—C8	118.86 (12)	N2—C8—S1	119.37 (11)
C7—N2—H2N	116.6 (13)	N3—C9—C10	108.91 (13)
C8—N2—H2N	114.2 (13)	N3—C9—H9A	109.9
C8—N3—C9	122.29 (13)	C10—C9—H9A	109.9
C8—N3—C12	126.02 (12)	N3—C9—H9B	109.9
C9—N3—C12	111.57 (12)	C10—C9—H9B	109.9
C2—C1—C6	122.68 (14)	H9A—C9—H9B	108.3
C2—C1—N1	118.30 (14)	O4—C10—C9	111.74 (15)
C6—C1—N1	119.02 (14)	O4—C10—H10A	109.3
C1—C2—C3	118.47 (14)	C9—C10—H10A	109.3
C1—C2—H2	120.8	O4—C10—H10B	109.3
C3—C2—H2	120.8	C9—C10—H10B	109.3
C2—C3—C4	120.54 (15)	H10A—C10—H10B	107.9
C2—C3—H3	119.7	O4—C11—C12	111.69 (13)
C4—C3—H3	119.7	O4—C11—H11B	109.3
C5—C4—C3	119.79 (14)	C12—C11—H11B	109.3
C5—C4—C7	123.23 (13)	O4—C11—H11A	109.3
C3—C4—C7	116.87 (13)	C12—C11—H11A	109.3
C4—C5—C6	120.20 (14)	H11B—C11—H11A	107.9
C4—C5—H5	119.9	N3—C12—C11	111.05 (13)
C6—C5—H5	119.9	N3—C12—H12B	109.4
C1—C6—C5	118.29 (14)	C11—C12—H12B	109.4
C1—C6—H6	120.9	N3—C12—H12A	109.4
C5—C6—H6	120.9	C11—C12—H12A	109.4
O3—C7—N2	122.60 (14)	H12B—C12—H12A	108.0
			
O1—N1—C1—C2	−173.39 (16)	C3—C4—C7—O3	24.2 (2)
O2—N1—C1—C2	6.9 (2)	C5—C4—C7—N2	27.0 (2)
O1—N1—C1—C6	5.8 (2)	C3—C4—C7—N2	−156.96 (15)
O2—N1—C1—C6	−173.87 (17)	C9—N3—C8—N2	−169.00 (14)
C6—C1—C2—C3	0.8 (3)	C12—N3—C8—N2	15.4 (2)
N1—C1—C2—C3	179.95 (15)	C9—N3—C8—S1	10.5 (2)
C1—C2—C3—C4	1.0 (3)	C12—N3—C8—S1	−165.17 (12)
C2—C3—C4—C5	−2.0 (2)	C7—N2—C8—N3	66.86 (18)
C2—C3—C4—C7	−178.23 (15)	C7—N2—C8—S1	−112.65 (14)
C3—C4—C5—C6	1.2 (2)	C8—N3—C9—C10	129.24 (16)
C7—C4—C5—C6	177.18 (14)	C12—N3—C9—C10	−54.55 (19)
C2—C1—C6—C5	−1.5 (2)	C11—O4—C10—C9	−60.10 (19)
N1—C1—C6—C5	179.29 (13)	N3—C9—C10—O4	58.40 (19)
C4—C5—C6—C1	0.5 (2)	C10—O4—C11—C12	57.60 (19)
C8—N2—C7—O3	−4.3 (2)	C8—N3—C12—C11	−130.55 (16)
C8—N2—C7—C4	176.88 (13)	C9—N3—C12—C11	53.41 (18)
C5—C4—C7—O3	−151.81 (16)	O4—C11—C12—N3	−54.50 (18)

### Hydrogen-bond geometry (Å, °)

**Table:**

Cg1 is the centroid of the C1–C6 ring.

**Table:**

*D*—H···*A*	*D*—H	H···*A*	*D*···*A*	*D*—H···*A*
N2—H2N···O4^i^	0.82 (2)	2.27 (2)	3.0947 (17)	178.1 (18)
C3—H3···O1^ii^	0.93	2.43	3.206 (2)	141
C6—H6···O3^iii^	0.93	2.65	3.377 (2)	135
C9—H9B···O4^iv^	0.97	2.67	3.590 (2)	159
C10—H10A···S1^v^	0.97	2.98	3.7913 (18)	142
C12—H12B···S1^vi^	0.97	2.97	3.7196 (18)	135
C2—H2···Cg1^vii^	0.93	3.45	3.682 (2)	83

Symmetry codes: (i) *x*−1, *y*+1, *z*; (ii) *x*+1, *y*, *z*; (iii) *x*−1, *y*, *z*; (iv) −*x*+2, −*y*, −*z*; (v) *x*+1, *y*−1, *z*; (vi) −*x*+1, −*y*+1, −*z*; (vii) −*x*, −*y*+2, −*z*+1.
